# Effects of Selected Metal Nanoparticles (Ag, ZnO, TiO_2_) on the Structure and Function of Reproductive Organs

**DOI:** 10.3390/toxics10080459

**Published:** 2022-08-08

**Authors:** Lucia Dianová, Filip Tirpák, Marko Halo, Tomáš Slanina, Martin Massányi, Robert Stawarz, Grzegorz Formicki, Roberto Madeddu, Peter Massányi

**Affiliations:** 1 Institute of Applied Biology, Faculty of Biotechnology and Food Sciences, Slovak University of Agriculture in Nitra, Tr. Andreja Hlinku 2, 949 76 Nitra, Slovakia; 2 Research Centre AgroBioTech, Slovak University of Agriculture in Nitra, Tr. Andreja Hlinku 2, 949 76 Nitra, Slovakia; 3 Institute of Biology, Pedagogical University of Kraków, ul. Podchorążych 2, 30-084 Kraków, Poland; 4 Department of Biomedical Sciences-Histology, University of Sassari, Viale San Pietro 43/B, 07100 Sassari, Italy

**Keywords:** nanoparticles, Ag, ZnO, TiO_2_, toxicity, reproductive system

## Abstract

Various studies have shown that the reproductive organs are highly sensitive to toxic elements found in the environment. Due to technological progress, the use of nanoparticles has become more common nowadays. Nanoparticles are used for drug delivery because their dimensions allow them to circulate throughout the body and enter directly into the cell. Antimicrobial properties are increasingly used in the manufacture of medical devices, textiles, food packaging, cosmetics, and other consumer products. Nanoparticles provide several benefits, but aspects related to their effects on living organisms and the environment are not well known. This review summarizes current in vivo, and in vitro animal studies focused on the evaluation of toxicity of selected metal nanoparticles (Ag, ZnO, TiO_2_) on male and female reproductive health. It can be concluded that higher concentrations of metal nanoparticles in the male reproductive system can cause a decrease in spermatozoa motility, viability and disruption of membrane integrity. Histopathological changes of the testicular epithelium, infiltration of inflammatory cells in the epididymis, and prostatic hyperplasia have been observed. Nanoparticles in the female reproductive system caused their accumulation in the ovaries and uterus. Metal nanoparticles most likely induce polycystic ovary syndrome and follicular atresia, inflammation, apoptosis, and necrosis also occurred.

## 1. Introduction

Reproduction is one of the most important biological processes responsible for the formation of a new organism. The main tasks of reproduction involve the transfer of genetic information to the offspring, as well as the development and maintenance of the species [1,2]. These functions are under the control of the reproductive system. This organ system shows significant differences in morphology, physiology, and behavior between males and females [3,4,5,6].

Toxic elements found in the environment have adverse effects on human reproduction as well as other living organisms. An increasing incidence of infertility over the past forty years has been observed. This negative trend can be associated with current lifestyles and industrial and technological progress [5,7,8]. In recent years, a significant increase in the use of nanoparticles (NPs) has been observed. Their miniature sizes imply that they have unique properties that help to improve existing technological processes and medical procedures [6,9]. Despite their many benefits, information about the impacts of NPs and nanomaterials on health and the environment is limited [10,11]. Some studies have reported their negative effects on male and female reproductive organs and gametes [12]. Available data have concluded that NPs can be toxic to testicular tissues as well as reduce spermatozoa quality and fertilization ability. They can act as endocrine disruptors in both males and females. Changes in sex hormone levels can contribute to inflammation, increased apoptosis, and ovarian damage in females [13].

NPs are small particles, with 1 to 100 nanometers in at least one dimension. NPs are relatively complex molecules that consist of three layers. The central parts of the NPs are usually responsible for their properties [14]. They are characterized by significantly different chemical and physical properties, such as greater surface areas, high reactivity, sensitivity, stability, magnetic, optical, thermal, and antimicrobial properties, or UV protection [15]. The descriptions of NPs depend on their shapes, size compositions, and other characteristics [10]. There are various groups of NPs; for example, polymeric and ceramic NPs or metal NPs and fullerenes [14]. NPs and nanomaterials provide numerous benefits and find applications in many research areas, including the mechanisms of drug supply, medical devices, bioimaging, food products and packing, cosmetics, the automotive industry, and other industries [14,16].

When NPs enter the body, they are absorbed and translocated to various organs, including the reproductive system, through the circulatory and lymphatic systems [16]. Epidemiological and occupational health studies involving animals and humans indicate the apparent toxicity of NPs. However, despite the increasing use of NPs, the amount of these studies are insufficient. The respiratory system is the primary route of exposure to NPs. Inhalation can lead to the translocation of metal NPs from the lungs to other organ systems. Oral exposure to NPs has been recorded when food containing NP additives was consumed. NPs can also enter the body through dermal contact or intravenously when they are used in the field of nanomedicine. Metal NPs and NPs containing metal oxides likely have the most notable cytotoxic effects. Cytotoxicity of metal NPs mainly leads to increased production of reactive oxygen species (ROS) and a decrease in cell viability [17,18,19,20]. NPs can easily cross the blood–brain and blood–testicular barriers. One of the main mechanisms of the antimicrobial activity of NPs is the production of ROS [21]. It is confirmed that elevated ROS levels cause oxidative stress (OS), which is associated with increased inflammatory activity, cell membrane damage, DNA damage, and other pathological processes [22,23,24].

The properties of metal NPs mainly depend on their composition, morphology, size, composition, and crystalline structure. They are also characterized by high surface activity, which is because NPs have a higher percentage of atoms on their surfaces [25]. The most used metal NPs in medicine, food, agriculture, and industry include Ag, ZnO, TiO_2_, CuO, CeO_2_, and FeO [26,27,28]. The reproductive toxicity of said NPs may be a public health problem due to the use of consumer products containing NPs or, unquestionably, due to an occupational disease during the production of these products [17]. This review discusses the effects of selected metal NPs (Ag, ZnO, TiO_2_) on the structures and functions of male and female reproductive organs.

## 2. Silver Nanoparticles (AgNPs)

AgNPs are metal NPs that are widely used in the manufacturing of various materials and products [29]. At present, they are among the most frequently used NPs, mainly due to their powerful antimicrobial effects. They are incorporated into various materials, such as textiles and plastics, as antibiotic agents, granting special added value to clothing, food packaging, toys, wound dressings, cosmetics, medical devices, electronic appliances, pharmaceuticals, and various other products [30,31,32]. Due to the frequent use of AgNPs in consumer products, despite several positive properties, it is inevitable to draw attention to the detrimental effects of exposure to these particles [30]. According to the Consumer Product Inventory (CPI), there are already 443 commercial products containing nanosilver on the market [33]. Ema et al. [30] have stated that occupational exposure is possibly more harmful than that of consumer or environmental exposure due to manipulation with pure forms of NPs in large measures.

Prolonged exposure to AgNPs can have a negative impact on cells at the molecular level. As with many xenobiotics, the adverse effects are the results of enhanced ROS production and the subsequent development of oxidative stress in the body [34]. According to previous in vitro studies, nanosilver can interact with certain types of enzymes, bind to cells and, thus, disrupt cellular processes. This can prevent cellular inflammation, mitochondrial dysfunction, cell-cycle arrest, apoptosis, or necrosis [32].

### 2.1. Effect of AgNPs on the Male Reproductive System

According to several studies, AgNPs may be responsible for the pathological processes of the male reproductive tract. A probable cause of nanosilver reproductive toxicity is excessive production of ROS [35]. It was found that after 28 days of oral exposure, AgNPs were distributed to the organs of rats in the following order—small intestine, stomach, kidneys, liver, brain, and lungs [36]. High concentrations of nanosilver were also found in the testicles and spleens [37].

Fathi et al. [38] conclude that AgNPs can have deleterious effects on spermatozoa quality and seminiferous tubules. Adult male Wistar rats (*n* = 28) were divided into four groups—three experimental and one control group. Experimental groups received 30, 125, and 300 mg/kg bw of AgNPs. NPs were injected into the epididymis and sacrificed after 28 days. The authors of this study observed how AgNPs may influence quality parameters of spermatozoa, chromatin integrity, and changes in testicular morphology and histology 28 days after injection. Results of the experiment showed a decrease in the number of spermatogonia, Leydig, and Sertoli cells in groups that received 125 and 300 mg/kg of AgNPs. A dose of 300 mg/kg bw decreased vitality and the number of rat spermatozoa. Results did not show any differences or abnormalities of spermatozoa chromatin [38].

Another experimental work described significantly lower relative weights of the testes and epididymides of rats after 7 days of exposure to 10 and 50 mg/kg bw AgNPs. Both concentrations of AgNPs had negative impacts on hormonal concentrations. Spherical nanosilver with a particle size of 100 nm caused a significant decrease in the concentration of testosterone, the follicle-stimulating hormone (FSH), and the luteinizing hormone (LH) after 7 and 28 days of AgNPs treatment [39]. All analyzed hormones had irreplaceable roles in the proper course of spermatogenesis [40]. This study also evaluated the effects of AgNPs on spermatozoa parameters. A decrease in motility, progressive motility, and velocity parameters of rat spermatozoa confirmed dose-dependent adverse effects of AgNPs. Results of the experiment showed higher concentrations of H_2_O_2_ and increased lipid peroxidation in the testes and epididymides dosed at 50 mg/kg. Activity involving catalase, superoxide dismutase, and reduced glutathione were also reduced. In the case of the histopathology of testes and epididymides, degenerative alterations at the cellular level were observed [39].

Previous research by Castellini et al. [41] showed partially opposite results to the research by Olugbodi et al. [39]. In this study, New Zealand White male rabbits were once injected with 0.6 mg/kg AgNPs (volume—2.0 mL) and then observed for 126 days. The authors described that exposure to AgNPs had no impact on body weight, the concentration of testosterone, or ejaculate volume. In the case of the spermatozoa and testes ultrastructure, there were no histopathological changes compared to the control group. There were no signs of degenerative processes in the morphology of spermatids, spermatocytes, spermatogonia, Leydig, and Sertoli cells. Nevertheless, AgNPs were present in the cytoplasm of Sertoli cells and the nucleus of spermatid [41].

In 2014, research results by Garcia et al. [42] pointed out the ability of AgNPs to alter and impair the physiological functions of Leydig cells, which may subsequently cause changes in testosterone levels in the testes. The study focused on the ability of short-term exposure to AgNPs to induce reproductive toxicity in CD1 male mice. Subjects repeatedly received an intravenous dose of AgNPs at a concentration of 1 mg/mL for 12 days. There were no changes in testicular weight or spermatozoa motility and concentration. Serum levels of LH and FSH were also unaffected, but changes occurred in testosterone concentrations. Moreover, 15 days after the initial exposure to AgNPs, there was a significant increase in testosterone levels in the testes. Histology confirmed changes in the Leydig cell size, epithelial morphology, and germ cell apoptosis [42].

Spherical AgNPs with an average diameter of 40 nm had an obvious dose-dependent negative impact on mice spermatozoa in vitro. Spermatozoa were collected from the ampulla of the vas deferens by the swim-up method from BDF1 mice aged 8 to 12 weeks. Spermatozoa were diluted in a medium supplemented with 0.1, 1, 10, and 50 μg/mL AgNPs and then incubated for 3 h. Concentrations of 10 and 50 μm/mL of AgNPs had observably negative impacts on the acrosome reaction. These concentrations were also responsible for morphological abnormalities of spermatozoa and their reduced viability. Via transmission electron microscopy, it was found that lower concentrations of AgNPs were localized mainly on the spermatozoa membrane, while higher concentrations were found in the head and the mitochondrial segment. Furthermore, AgNP-treated spermatozoa compromised oocyte fertilization and embryo development [43].

The intention of the study by de Brito et al. [44] was to develop contraception for males using nanotechnology. In this experiment, 220 μL of AgNP solution at a concentration of 0.46 μg/mL was administered by intratesticular injection into both testes of Wistar rats. Subsequently, the rats were euthanized 7, 14, 28, and 56 days after injection. The result was a reduction in the percentage of spermatozoa motility. There were also changes in the structure and morphology in the middle part of the spermatozoa head [44].

As Zapór [45] described, Sertoli cells are involved in the formation of the blood–testis barrier. The effect of 10, 40, and 100 nm AgNPs on the Sertoli cell line (15P-1) was analyzed. AgNPs with sizes < 10 nm, were found to be prone to the formation of aggregates and have a stronger cytotoxic effect than larger NPs. In the case of Sertoli cells, AgNPs with sizes < 10 nm, were responsible for increased lipid peroxidation and overproduction of ROS, which caused subsequent DNA damage [45].

The most significant alterations of silver nanoparticles related to male reproductive organs are summarized in Table 1.

### 2.2. Effect of AgNPs on the Female Reproductive System

Due to the growing possibilities of using NPs in science, medicine, and everyday life, the aim of many studies is to assess their impact on the functions of the female reproductive system. During the intravenous administration of 30 nm silver nanospheres to laboratory mice, it has been found that the meiotic maturation of oocytes is impaired; increased necrosis and apoptosis of follicular cells were also detected. These pathological changes occurred at doses of 2 and 4 mg/kg of AgNPs administered 10 times. An increase in the index of contractility of the uterus was observed at a dose of 4 mg/kg of AgNPs administered 5 times [46].

A recent study by Katarzyńska-Banasik et al. [37] investigated the effects of the oral administration of AgNPs on ovarian steroidogenesis and the concentration of steroid hormones in the blood plasma of Hy-Line Brown hens. It also analyzed the effects of triiodothyronine and thyroxine, which are thyroid hormones that affect ovarian steroidogenesis. The results confirm that AgNPs are able to indirectly disrupt ovarian steroidogenesis and affect the metabolism of thyroid hormones. Oral exposure to nanosilver increased concentrations of triiodothyronine in blood plasma. According to the study, prehierarchical follicles seem to be more targeted by AgNPs than preovulatory ones [37].

Polycystic ovary syndrome (PCOS) is usually the result of hormonal imbalance. Approximately 5–20% of women of reproductive age suffer from this disease. Alwan and Al-Saeed [47] examined the effect of AgNPs, biosynthesized by using Cinnamomum zeylanicum bark extract, on the fertility of rats with induced PCOS. The first of the three experimental groups was treated with a dose of 200 mg/kg of methanol extract Cinnamomum zeylanicum, the second group with 50 mg/kg of metformin, and the third with 3.53 mg/kg of biosynthesized AgNPs for 30 days. The study evaluated the levels of sex hormones (estradiol, progesterone, testosterone, LH, and FSH). According to the results of the experiment, biosynthesized AgNPs may affect the pathological process of PCOS by regulating the hormones involved in the development of the disease. After 30 days of treatment with AgNPs, ovarian tissues were regenerated, and female fertility improved from 25% to 100% [47].

The effect of NPs on cells, tissues, and organs is dose- and time-dependent. This claim is also supported by the research by Luaibi and Qassim [48]. The authors studied 60 female Sprague–Dawley rats to examine the effects of AgNPs on sex hormone levels (FSH, LH, progesterone, estrogen), ovarian functions, and histology, depending on the dose administered and the time of exposure. Three groups of animals were exposed to AgNPs for 10, 20, and 30 days. At each time point, the subgroups were treated with 12.5, 25, and 50 mg/kg of AgNPs (20–30 nm) by intraperitoneal injection. A significant increase compared to the control group was observed for estrogen levels in the shortest time interval (10 days), at all used concentrations of AgNPs. After 20 days, serum estrogen levels increased to 12.5 mg/kg, while concentrations of 25 and 50 mg/kg decreased. As a result of exposure to AgNPs for 30 days, estrogen levels were demonstrably reduced at all concentrations monitored. Histology showed an increase in ovarian weight after 30 days and a dose of 50 mg/kg AgNPs [48].

The most significant alterations of silver nanoparticles related to female reproductive organs are summarized in Table 2.

## 3. Zinc Oxide Nanoparticles (ZnO NPs)

ZnO NPs have excellent semiconductor, optical, antibacterial, antifungal, and other properties. Due to these properties, they are becoming more frequently used [49]. Their high optical absorption in the UVA and UVB range is especially noteworthy. ZnO NPs are therefore used in cosmetics to produce sunscreens [50]. The antimicrobial activities of these NPs are also attracting attention. The nano-dimensional zinc oxide can enter the cell and interact with the cell membrane and/or the bacterial nucleus. According to several studies, ZnO NPs are not considered toxic for human cells; they are described as biocompatible. Their antimicrobial effects are also applied in the food industry, although the mechanisms of their actions, as well as other NPs, remain under discussion [51]. ZnO NPs are currently components of 38 consumer products [33].

### 3.1. Effects of ZnO NPs on Male Reproductive System

Zinc is one of the essential trace elements in the human body. Since this element cannot be stored in the body, it is necessary to ensure its intake by food. In the case of male reproduction, it plays an important role in ensuring hormonal balance, spermatogenesis, regulation of capacitation, acrosome reaction, and maintaining the lining of male reproductive organs. Zinc deficiency is often responsible for abnormal spermatozoa morphology and adversely affects serum testosterone levels [52].

Although zinc is a biogenic element, NPs of this element can stimulate damage to various tissues and organs in the body. Thus, Radhi et al. [53] investigated the effects of ZnO NPs on the reproductive systems of male albino mice. The quality of the spermatozoa in the epididymal tail and the weight of the reproductive organs (testicles, epididymides, and accessory gonads) were evaluated. Individual groups of male albino mice were treated with a dose of 100 or 200 mg/kg ZnO NPs for 7 or 14 days. ZnO NPs reduced the weight of the testes and epididymides with an increasing dose and exposure time, especially at 200 mg/kg for 14 days. In contrast, the weight of the prostate and seminal vesicles increased. The percentage of damaged spermatozoa in the epididymis of mice in all observed groups also increased [53].

Several studies are available that look at the protective effects of ZnO NPs against reproductive organ damage caused by the side effects of some anti-cancer drugs but also other toxic substances, such as nicotine [54,55,56]. Doxorubicin is an anthracycline that is used in chemotherapy because it is effective against a wide range of cancers. Carvalho et al. [57] described this antibiotic as a “double-edged sword” because it very often causes non-target tissue damage, which ultimately complicates the cancer treatment itself [57]. In order to alleviate the toxic side effects of this treatment, El-Maddawy et al. [54] decided to assess the possible protective effects of ZnO NPs against doxorubicin-induced testicular toxicity in male rats. A total of 40 Wistar rats were divided into 4 groups as follows: (1) control group; (2) 3 mg/kg ZnO NPs; (3) 6 mg/kg doxorubicin; (4) 3 mg/kg ZnO NPs + 6 mg/kg doxorubicin. Significant negative effects on spermatozoa motility and viability percentage, epididymal sperm count, and spermatozoa morphology were observed in the doxorubicin-treated group of rats. Reproductive system weight loss has also been reported. ZnO NPs did not cause any adverse effects on the endpoints, while in combination with doxorubicin, it reduced its negative side effects on the reproductive system of rats [54]. Positive effects of ZnO NPs have also been reported in combination with nicotine [56] and another anticancer drug—cyclophosphamide [54].

Another study that assessed the effects of ZnO NPs on mouse spermatogenesis concluded that these NPs may cause reproductive toxicity in male mice. Experimental groups received doses of ZnO NPs 5, 50, and 300 mg/kg for 35 days. According to the obtained results, it was found that high doses of ZnO NPs (50 and 300 mg/kg) caused histopathological changes, such as sloughing of immature germ cells and vacuolization of the seminiferous epithelium [58].

Disruption of DNA integrity is a known problem in the cryopreservation of sperm of any animal species. Isaac et al. [59] assessed the effects of ZnO NPs as additives to cryopreservation media on human sperm damage due to freezing and thawing. They found that the percentage of sperm motility was not significantly increased but showed a significant reduction in the number of DNA-damaged sperm and decreased malondialdehyde (MDA) levels [59]. MDA is characterized as an indicator of lipid peroxidation [60]. Thus, the authors of the study state that the addition of ZnO NPs to cryopreservation media has a beneficial effect [59].

Halo Jr. et al. [61] investigated the effects of ZnO NPs on rabbit spermatozoa viability and motility in vitro. Ejaculates were collected from eight New Zealand rabbits. Ejaculates were then diluted with ZnO NPs at 6, 12, 24, 49, 98, 195, and 391 mg/mL, and subsequently analyzed at 0, 1, 2, and 3 h at 37 °C. The results showed that ZnO NPs at higher concentrations had a negative impact on cell membrane integrity and spermatozoa viability. Motility and progressive motility were also significantly reduced. As with other studies, the authors of this study agree that spermatotoxicity of ZnO NPs is dose- and time-dependent [61].

The most significant alterations of zinc oxide nanoparticles related to male reproductive organs are summarized in Table 3.

### 3.2. Effects of ZnO NPs on the Female Reproductive System

Higher doses of ZnO NPs induce reproductive toxicity in females as well as males, as confirmed by an experimental study by Mohammad Hosseini et al. [62], which aimed to assess the subchronic effects of different doses of ZnO NPs on the reproductive organs of female Wistar rats. Rats were exposed twice a week for one month to doses of 4, 8, 25, 50, 100, and 200 mg/kg ZnO NPs (size 10–30 nm). Blood samples were then analyzed and the ovaries and uterus were collected for histopathological analysis. The authors found that follicular cysts, inflammatory lesions, hyperemia, and corpus luteum increased in the ovaries, while epithelial destruction and endometrial gland hyperplasia were present in the uterus. The number of pathological findings in the uterus and ovaries increased depending on the dose of NPs. There was also a significant increase in progesterone and estrogen levels at concentrations of 4 mg/kg ZnO NPs and a consequent decrease in serum concentrations of both sex hormones at concentrations of 200 mg/kg ZnO NPs [62].

Another study reported adverse effects of ZnO NPs on female reproductive health. The authors of that study found that all markers of apoptosis (caspase 3, caspase 9, Bcl, and Bax) were increased by ZnO NPs in all analyzed groups. Follicle and ovarian tissue degeneration also occurred in all groups. However, in this experiment, it was also found that l-arginine was able to help tissue regeneration and act preventively against damage caused by ZnO NPs. In this experiment, two groups of Wistar albino rats were administered 100 and 200 mg/kg ZnO NPs and the other two groups received 100 and 200 mg/kg ZnO NPs + 1.3 g/kg l-arginine once daily for 21 days [63].

ZnO NPs are widely used in consumer products, such as food packaging, cosmetics, and more. Therefore, the authors of the following study decided on oral administration of the ZnO NPs suspension at a concentration of 100 mg/kg to mice. In this experiment, similar results were recorded as in the experiment performed by the aforementioned Efendic et al. [63]. Pathological and inflammatory cells were present in the ovaries of mice treated with ZnO NPs. Accumulation of NPs in the uterus and ovaries has also been reported and structural changes in the myometrium have also occurred [64].

The most significant alterations of zinc oxide nanoparticles related to female reproductive organs are summarized in Table 4.

## 4. Titanium Dioxide Nanoparticles (TiO_2_ NPs)

TiO_2_ NPs are part of many consumer products [65]. According to CPI, TiO_2_ NPs are currently contained in 93 consumer products [33]. TiO_2_ NPs may be found in foods, personal care products, sunscreens, topcoats, and more [65]. Candies or chewing gums are considered the food with the highest content of TiO_2_. By using these products, NPs also seep into wastewater and the environment [66]. Due to their photoactive properties, TiO_2_ NPs have come to the attention of many scientists. These NPs can produce a variety of reactive oxygen species after being illuminated by UV light in an aqueous environment. In this way, it is possible to induce cell death, which has been used in medicine in the form of photodynamic therapy. This therapy is used to treat many diseases, including cancer and psoriasis [67].

### 4.1. Effect of TiO_2_ NPs on the Male Reproductive System

A study by Azmy et al. [68] focused on the reproductive toxicity of TiO_2_ NPs in male Albino rats. A total of 40 rats were orally administered a dose of 100 mg/kg of TiO_2_ NPs daily. The rats were divided into two groups. The first group (*n* = 20) was sacrificed after 4 weeks and the rest after 8 weeks from the beginning of the experiment. The results showed that after 4 weeks the relative organ weights of the testes, epididymides, and seminal vesicles decreased significantly, and after 8 weeks, the weight of the testes and epididymides significantly reduced. In both groups, there was a reduction in the percentage of sperm motility and a reduction in their concentrations. After 4 and 8 weeks, testosterone levels were reduced and morphological abnormalities of spermatozoa, such as deformed and detached heads and curved and coiled tails occurred. A histopathological examination was performed after 8 weeks. Exposure to TiO_2_ NPs caused edema and sloughing of the testicular epithelium, inflammatory cell infiltration, congestion of the epididymis, edema, congestion, hyperplasia of the prostate, and widespread seminal vesicle congestion [68].

The reproductive toxicity of TiO_2_ NPs was also confirmed by the results of the experiment performed by Song et al. [69]. A total of 60 male ICR mice were intragastrically treated with 0, 10, 50, and 100 mg·kg^−1^ TiO_2_ NPs daily for 28 days. As in the research by Azmy et al. [68] and Shahin and Mohamed [70], the experimental results also showed that TiO_2_ NPs are responsible for reducing spermatozoa quality. When the dose of TiO_2_ NPs reached 50 mg/kg, the percentage of motility and various morphological abnormalities in spermatozoa decreased. Superoxide dismutase is an enzyme that is considered a major scavenger of oxygen free radicals [71]. At a dose of 100 mg/kg of TiO_2_ NPs, the activity of this enzyme in testes was significantly reduced. At a dose of 50 mg·kg^−1^, an increase in MDA concentration was observed [69].

From the point of view of nanotoxicity, it is interesting to investigate the interaction of several NPs together. The authors of the study, who investigated co-exposure to TiO_2_ and ZnO NPs as well as ZnO and TiO_2_ separately, came up with this idea. In this experiment, Ogunsuyi et al. [72] divided 65 Swiss mice into three groups. Each group was exposed to the same concentrations of NPs (9.38, 18.75, 37.5, 75 mg/kg) but the first group was treated with TiO_2_ NPs, the second ZnO NPs, and the third group was exposed to a 1:1 combination of ZnO and TiO_2_ NPs. The NPs were administered daily for 35 days by intraperitoneal injection. The results of the research showed a significant decrease in sperm motility and concentration and an increase in the number of spermatozoa with morphological damage. A larger number of morphological abnormalities in spermatozoa was observed in the group exposed to a mixture of ZnO and TiO_2_ NPs. In all three groups, the LH level was also reduced, while the testosterone level increased but only by the effect of ZnO NPs and their mixture with TiO_2_. Histology revealed deviations from normal testicular morphology. It was confirmed that TiO_2_ NPs, similar to the studies by Song et al. [69] and Azmy et al. [68], caused histopathological changes in the testicular tissue. In the case of a combination of ZnO and TiO_2_, according to the results of this study, they have a synergic effect and induce testicular damage [72].

Gao et al. [73], in their study, revealed that TiO_2_ NPs can cause changes in the expressions of genes that are involved in the process of spermatogenesis and sex hormone metabolism. They found that intragastric administration of TiO_2_ NPs at 2.5, 5, and 10 mg/kg for 90 days induced accumulation of NPs in the testes, which caused changes in sex hormone levels, testicular lesions, and morphological damage to spermatozoa [73].

The most significant alterations of titanium dioxide nanoparticles related to male reproductive organs are summarized in Table 5.

### 4.2. Effect of TiO_2_ NPs on Female Reproductive System

TiO_2_ NPs are able to accumulate in the lungs, brain, spleen, kidneys, and liver. It is questionable whether TiO_2_ NPs can also be distributed and accumulated in the ovaries and subsequently affect female fertility [74]. The research results by Gao et al. [75] support the hypothesis that TiO_2_ NPs can affect ovarian functions through the regulation of some ovarian genes. Female ICR mice (*n* = 150) received 10 mg/kg of TiO_2_ NPs intragastrically for 90 days. The results showed that this dose of NPs can accumulate in the ovaries and subsequently cause ovarian damage. Furthermore, NPs can cause hormonal imbalances, reduce fertility, disrupt the distribution of mineral elements, and be responsible for oxidative stress [75].

The following study also demonstrated that exposure to TiO_2_ NPs can lead to reduced fertility in females and slower embryonic development. A total of 54 female NMRI mice were orally administered 100 mg/kg of TiO_2_ NP solution daily for 5 weeks. Based on the results, the authors showed that MDA and estrogen levels were significantly increased. There was also a reduction in the oocyte count and fertilization rate. Histology has shown pathological changes and the formation of cysts in the ovaries. Developmental disorders, degeneration, and decreased ovarian follicle counts were also present [76].

The above changes in the form of ovarian damage and reduced fertility due to TiO_2_ NPs are also confirmed by the findings of Zhao et al. [77]. The effects of these particles on sex hormone levels, pro-inflammatory cytokine expression, follicular atresia, and ovarian damage after intragastric administration of 2.5, 5, and 10 mg/kg of TiO_2_ NPs daily for 90 days to female mice were evaluated. As in previous studies, the relative organ weights of the ovaries and fertility were reduced. Changes in serum sex hormones, atresia, inflammation, and follicular necrosis were also observed. Decreased expressions of the LH receptor, insulin-like growth factor-1, and inhibin-α occurred in the ovaries. In contrast, there were increased expressions of the epidermal growth factor, tissue plasminogen activator, and inflammatory cytokines, specifically interleukin-1β, interleukin-6, and tumor necrosis factor-α [77].

The most significant alterations of titanium oxide nanoparticles related to female reproductive organs are summarized in Table 6.

## 5. Conclusions

Based on the research presented in this review, it can be concluded that metal nanoparticles (Ag, ZnO, TiO_2_), especially at higher concentrations, cause reproductive toxicity in both females and males. Their negative effects are most often manifested in the disruption of sex hormone levels, the development of oxidative stress, inflammation, and histopathological changes in the reproductive organs. Despite all of the negative effects, a positive effect of low concentrations of ZnO NPs was also observed. All nanoparticles described were found to be strongly time- and dose-dependent. Since the mechanisms of action of metal nanoparticles on living organisms have been poorly investigated, further research in this area is needed in the future. It is necessary to responsibly consider the need for nanoparticle usage in consumer products. It is equally important to pay attention to the fact that, due to the lack of epidemiological studies on the effects of nanoparticles on human health, working with nanomaterials can pose serious health risks for workers. This review presents the possible risks regarding reproduction and suggests the responsible use of nanoparticles, due to their numerous positive, but also negative effects.

## Figures and Tables

**Table 1:** Alterations in the male reproductive system caused by AgNPs.

Administration/Dose/Species/Size of NPs	Changes	References
Single injection to the epididymis 30, 125, 300 mg/kg sacrificed after 28 days Wistar rats Not specified	- Decrease in the number of spermatogonia, Leydig, and Sertoli cells - Decreased vitality and number of spermatozoa	Fathi et al., 2019 [38]
Subcutaneous injections 10, 50 mg/kg/day; 7, 28 days Wistar rats 100 nm	- Lower relative weights of the testes and epididymides - Decrease in the level of LH, FSH, and testosterone - Decrease of the total, progressive motility, and velocity - Reduced catalase, superoxide dismutase, and reduced glutathione	Olugbodi et al., 2020 [39]
Single intratesticular injection 220 μL (c = 0.46 μg/mL); sacrificed after 7, 14, 18, 56 days Wistar rats Not specified	- Reversible reduction of spermatozoa motility at all monitored time intervals - Changes in the structure and morphology in the middle part of the spermatozoa head	de Brito et al., 2020 [44]
Intravenous injection 1 mg/mL/day; 12 days CD1 mice not specified	- Increased testosterone levels in the testes - Histological changes in Leydig cell size, epithelial morphology, and germ cell apoptosis	Garcia et al., 2014 [42]
Single intratesticular injection 0.6 mg/kg; observed 126 days; New Zealand White rabbits not specified	- AgNPs were present in the cytoplasm of Sertoli cells and the nucleus of spermatid	Castellini et al., 2014 [41]

**Table 2:** Alterations in the female reproductive system caused by AgNPs.

Administration/Dose/Species/Size of NPs	Changes	References
Intravenous injection 2, 4 mg/kg; 10× Mice 30 nm	- Increased necrosis and apoptosis of follicular cells - Increased index of contractility of the uterus	Lytvynenko et al., 2017 [46]
Per os not specified Hy-Line Brown hens not specified	- Disruption of ovarian steroidogenesis	Katarzyńska-Banasik et al., 2021 [37]
Intraperitoneal injection 3.53 mg/kg/day; 30 days of biosynthesized AgNPs (with Cinnamomum zeylanicum bark extract) rats with PCOS not specified	- Ovarian tissues were regenerated, fertility improved	Alwan and Al-Saeed, 2021 [47]
Intraperitoneal injection 12.5, 25, 50 mg/kg/day; 10, 20, 30 days Sprague–Dawley rats 20–30 nm	- 12.5 mg/kg increased serum estrogen levels - 25 and 50 mg/kg decreased serum estrogen levels	Luaibi and Qassim, 2017 [48]

**Table 3:** Alterations in the male reproductive system caused by ZnO NPs.

Administration/Dose/Species/Size of NPs	Changes	References
Per os 100, 200 mg/kg/day; 7, 14 days Albino mice not specified	- Reduced weight of testes and epididymides - Increased weight of the prostate and seminal vesicles - Increased percentage of damaged sperm	Radhi et al., 2019 [53]
Per os 5, 50, 300 mg/kg/day; 35 days NMRI mice not specified	-50 and 300 mg/kg caused histopathological changes—sloughing of immature germ cells and vacuolization of the seminiferous epithelium	Talebi et al., 2013 [58]
In vitro 6–391 mg/mL; 0–3 h New Zealand rabbits <100 nm	-Higher concentrations—negative impact on cell membrane integrity -Reduced viability, motility, and progressive motility	Halo Jr. et al., 2021 [61]
in vitro 10–200 µg/mL; 2 weeks (cryopreservation) Human Not specified	- Lower sperm chromatin damage - Decreased malondialdehyde (MDA) level	Isaac et al., 2017 [59]

**Table 4:** Alterations in the female reproductive system caused by ZnO NPs.

Administration/Dose/Species/Size of NPs	Changes	References
Intravenous injection 4–200 mg/kg/twice a week; 1 month Wistar rats 10–30 nm	- Ovaries: follicular cysts, inflammatory lesions, hyperemia, and corpus luteum increased - Uterus: epithelial destruction and endometrial gland hyperplasia	Mohammad Hosseini et al., 2019 [62]
Intraperitoneal injection 100, 200 mg/kg/day; 21 days Wistar albino rats 10–30 nm	- Increased markers of apoptosis (caspase 3, caspase 9, Bcl, Bax) - Follicle and ovarian tissue degeneration	Efendic et al., 2022 [63]
Per os 100 mg/kg/day; 3 days Kunming mice not specified	- Inflammatory cells in ovaries - Accumulation of NPs in uterus and ovaries - Structural changes in the myometrium	Kuang et al., 2021 [64]

**Table 5:** Alterations in the male reproductive system caused by TiO_2_ NPs.

Administration/Dose/Species/Size of NPs	Changes	References
Per os 100 mg/kg/day; 8 weeks Wistar albino rats not specified	- Reduced weight of the testes and epididymides - Decreased sperm count and motility - Reduced levels of testosterone - Deformed and detached heads; curved and coiled tails of spermatozoa - Edema and sloughing of the testicular epithelium, inflammatory cell infiltration - Hyperplasia of the prostate	Azmy et al., 2015 [68]
Per os 0–100 mg/kg/day; 28 days ICR mice not specified	- Decreased motility - Morphological abnormalities of spermatozoa - Reduced activity of SOD - Increased MDA levels	Song et al. 2017 [69]
Intraperitoneal injection 9.38–75 mg/kg/day; 35 days Swiss mice not specified	- Decrease in spermatozoa motility and concentration - Increased number of spermatozoa with morphological damage - Histopathological changes in testicular	Ogunsuyi et al., 2020 [72]
Intragastric administration 2.5–10 mg/kg/day; 90 days Albino mice not specified	- Accumulation of NPs in the testes - Changes in sex hormone levels - Changes in the expression of genes that are involved in the process of spermatogenesis - Testicular lesions and morphological spermatozoa damage	Gao et al., 2013 [73]

**Table 6:** Alterations in the female reproductive system caused by TiO_2_ NPs.

Administration/Dose/Species/Size of NPs	Changes	References
Intragastric administration 10 mg/kg/day; 90 days ICR mice not specified	- Accumulation in the ovaries - Hormonal imbalances - Reduced fertility - Disrupted distribution of mineral elements - Oxidative stress	Gao et al., 2012 [75]
Per os 100 mg/kg/day; 5 weeks NMRI mice not specified	- Increased MDA and estrogen levels - Reduction in oocyte count and fertilization rate - Cysts in the ovaries	Karimipour et al., 2018 [76]
Intragastric administration 2.5–10 mg/kg/day; 90 days ICR mice not specified	- Follicular atresia, inflammation, and necrosis - Increased expression of epidermal growth factor, tissue plasminogen activator, interleukin-1β, interleukin-6, tumor necrosis factor-α	Zhao et al., 2013 [77]

## Data Availability

Not applicable.
